# Plastome Reduction in the Only Parasitic Gymnosperm *Parasitaxus* Is Due to Losses of Photosynthesis but Not Housekeeping Genes and Apparently Involves the Secondary Gain of a Large Inverted Repeat

**DOI:** 10.1093/gbe/evz187

**Published:** 2019-08-27

**Authors:** Xiao-Jian Qu, Shou-Jin Fan, Susann Wicke, Ting-Shuang Yi

**Affiliations:** 1 Key Lab of Plant Stress Research, College of Life Sciences, Shandong Normal University, Ji’nan, Shandong, China; 2 Germplasm Bank of Wild Species, Kunming Institute of Botany, Chinese Academy of Sciences, Kunming, Yunnan, China; 3 Institute for Evolution and Biodiversity, University of Muenster, Germany

**Keywords:** plastome, parasitism, mycoheterotrophy, gene loss, *Parasitaxus*

## Abstract

Plastid genomes (plastomes) of parasitic plants undergo dramatic reductions as the need for photosynthesis relaxes. Here, we report the plastome of the only known heterotrophic gymnosperm *Parasitaxus usta* (Podocarpaceae). With 68 unique genes, of which 33 encode proteins, 31 tRNAs, and four rRNAs in a plastome of 85.3-kb length, *Parasitaxus* has both the smallest and the functionally least capable plastid genome of gymnosperms. Although the heterotroph retains chlorophyll, all genes for photosynthesis are physically or functionally lost, making photosynthetic energy gain impossible. The pseudogenization of the three plastome-encoded light-independent chlorophyll biosynthesis genes *chlB*, *chlL*, and *chlN* implies that *Parasitaxus* relies on either only the light-dependent chlorophyll biosynthesis pathway or another regulation system. Nesting within a group of gymnosperms known for the absence of the large inverted repeat regions (IRs), another unusual feature of the *Parasitaxus* plastome is the existence of a 9,256-bp long IR. Its short length and a gene composition that completely differs from those of IR-containing gymnosperms together suggest a regain of this critical, plastome structure-stabilizing feature. In sum, our findings highlight the particular path of lifestyle-associated reductive plastome evolution, where structural features might provide additional cues of a continued selection for plastome maintenance.

## Introduction

The plastid’s principal function in photosynthesis is reflected in its semiautonomous genome (plastome) that encodes many components for the photosynthetic machinery (Palmer 1985; Wicke et al. 2011). Seed plant plastomes often exhibit a quadripartite structure with a conserved pair of large inverted repeats (IRs; 10–30 kb) separated by a large and a small single-copy (LSC and SSC) region, respectively (Jansen and Ruhlman 2012). Because of the selection pressure on photosynthesis-related elements, plastomes usually have a highly conserved gene content (115–160 genes) and gene order (Ruhlman and Jansen 2014), although notable exceptions are known (e.g., Guisinger et al. 2011; Chaw et al. 2018).

The most aberrant plastid genome structures pertain to heterotrophic (parasitic) plants, which have experienced parallel gene losses as they independently transitioned into a nonphotosynthetic lifestyle (Wicke and Naumann 2018). Plants can abandon photosynthesis as they have gained the ability to feed on other plants (Westwood et al. 2010). To obtain nutrients, so-called haustorial parasites develop a specialized feeding organ to tap into another plant’s vascular system, whereas mycoheterotrophic plants epiparasitize fungal networks. However, our knowledge of the progression of the heterotrophy-associated reductive plastome evolution is limited to flowering plants.

Podocarpaceae is the largest family of cupressophytes (Farjon 2001; Christenhusz and Byng 2016) and the only one in which a heterotrophic gymnosperm has evolved. *Parasitaxus usta* from New Caledonia establishes an obligate and direct fusion with the roots of one of its family members, *Falcatifolium taxoides*, which, as host, supplies the parasite with nutrients (Delaubenfels 1959). The plastids of *Parasitaxus* contain high contents of chlorophyll *a* and *b* but perform no photosynthetic electron transport (Feild and Brodribb 2005). The heterotrophic gymnosperm lacks roots, and it has no haustorium that usually characterizes haustorial parasitic flowering plants. Together, the presence of fungal hyphae at the parasite–host junction, the direct plant–plant connection, and carbon isotope ratios indicative of additional nutrient uptake from a fungus suggest that *Parasitaxus* “presents a unique physiological chimera” of haustorial parasites and mycoheterotrophic plants (Feild and Brodribb 2005, p. 1316). The heterotroph’s physiological uniqueness, therefore, might also exhibit uniqueness in reductive plastome evolution.

Here, we sequenced, assembled, and annotated the complete plastomes of *Parasitaxus usta* and its photosynthetic relative *Manoao colensoi*. It was the aim of our study to infer whether the heterotrophic mode of *Parasitaxus* follows the predicted course of heterotrophy-associated plastome reduction that is characteristic of nonphotosynthetic land plants. We focused our analyses on changes of the plastid coding capacity of *Parasitaxus* and found that its degree of physical and functional reduction, as well as its structural evolution, is exceptionally different from those of other heterotrophs.

## Materials and Methods

For plastid genome reconstruction via genome-skimming, we isolated total genomic DNA from fresh leaves of *Manoao* (deposited at Herbarium at the Royal Botanic Garden, Edinburgh, voucher no. 19842513) using the CTAB method (Doyle and Doyle 1987) and obtained a DNA sample of *Parasitaxus* from the DNA Bank of the Royal Botanical Gardens Kew (DNA Bank ID: 37534). Approximately, 1 μg of total DNA each was subjected to library construction with the NEBNext DNA Library Prep Kit and Covaris-based fragmentation to 650 bp before sequencing in 150-bp paired-end mode was carried out on an *Illumina* MiSeq. To obtain a sufficient postlibrary quantity for sequencing, 14 PCR cycles with the Q5 High-Fidelity DNA polymerase were necessary for *Parasitaxus* compared with eight for *Manoao*.

We assembled the plastomes using both the *Organelle Genome Assembler* (OGA) pipeline (Qu 2019) and Spades v3.13.0 (Bankevich et al. 2012), the latter with the “careful”-option and k-mers of 61, 81, 101, and 121. To validate the plastome assembly, we mapped all paired reads to the assembled plastomes with Bowtie v2.3.2 (Langmead and Salzberg 2012) with the local-sensitive option (-D 15 -R 2 -N 0 -L 20 -i S,1,0.75). We paid particular attention to the boundaries of inverted repeat to single-copy regions. For this, we checked read stacks by eye in Geneious v8.0.2 (https://www.geneious.com). An initial annotation by the *Plastid Genome Annotator* (PGA) (Qu et al. 2019) was refined by manual corrections in Geneious v8.0.2. We classified a gene as a pseudogene if its reading frame was truncated (incl. due to a premature stop codon) or frameshifted compared with nonparasitic Podocarpaceae (Wicke et al. 2016; Wicke and Naumann 2018). To verify whether genes not detected during initial annotation were really absent from the *Parasitaxus* plastome, we mapped again all its read data to the finished assembly of its close relative *Manoao* using Bowtie v2.3.2. Additionally, we used BlastN and intact orthologs from *Manoao* as queries to search the contig pool of *Parasitaxus*. Pseudogene candidates of *Parasitaxus* were double checked by aligning them with their functional equivalents of *Manoao*. Physical plastome maps were drawn with OGDRAW v1.2 (Lohse et al. 2013). The annotated plastomes are deposited in GenBank under accession numbers MN016935 and MN016936.

For a comparative phylogenomic analysis, we downloaded the plastomes of all previously sequenced Podocarpaceae species from GenBank plus *Zamia furfuraceae* as an outgroup (supplementary table S1, Supplementary Material online). We extracted their coding sequences and merged these data with those of the newly sequenced species. After building an alignment consisting of 82 protein-coding, 31 tRNA, and 4 rRNA genes with MAFFT v7.313 (Katoh and Standley 2013) under the FFT-NS-i x1000 option, we reconstructed phylogenetic relationships under the maximum likelihood (ML) paradigm with RAxML v8.2.10 (Stamatakis 2014), using the GTR-Γ substitution model and assessed tree robustness by 1,000 rapid bootstrap replicates. To assure that the ML inferences were not affected by long gaps caused by missing genes in *Parasitaxus*, we repeated this phylogenetic analysis on a reduced data set consisting of only the commonly present genes.

To determine plastome rearrangements, we determined the set of locally collinear blocks (LCBs) and inferred potential breakpoints through whole-plastome alignments of all 15 Podocarpaceae species and *Zamia* using progressiveMAUVE with default settings (Darling et al. 2010). Before genome alignment, we removed one IR copy from plastomes with large inverted repeats because such large duplicated regions hamper plastome alignments (Wicke et al. 2013). Additionally, plastid genomic repeat content was inferred from the number and length of forward, reverse, complement, and inverted repeats in a REPuter analysis (Kurtz et al. 2001), using a minimum length of 20 bp, a Hamming distance of 3, and a maximum *e*-value of 10^−3^. Tandem repeats in the plastome of *Parasitaxus* were identified using Phobos v3.3.12 (http://www.rub.de/ecoevo/cm/cm_phobos.htm; last accessed May 1, 2019), with a minimum length of 10 bp for perfect repeats and a repeat unit length of 2–50 bp. We used BayesTraits v3.0.1 and random walk models to analyze the plastid repeat history (Pagel et al. 2004). Testing for correlations between repeat density, the presence of an IR, number of rearrangements, and the transition to a nonphotosynthetic lifestyle were based on likelihood ratio tests (LRTs) between a free model that estimates the correlation between any two traits and one with no correlation assumed (Wicke et al. 2013). The number of rearrangements was reconstructed by a permutation analysis of locally collinear blocks over the fixed phylogenetic tree using MGR v2.01 (Tesler 2002; Röschenbleck et al. 2017).

Possible heterotrophy-associated changes of molecular evolution were evaluated from the distribution of selection regimes in Podocarpaceae. To this end, we analyzed a combined data set of all commonly present 33 protein-coding genes using a genetic algorithm approach (Kosakovsky Pond and Frost 2005) with the MPI-enabled HyPhy suite v2 (Kosakovsky Pond et al. 2005). This analysis models classes of nonsynonymous to synonymous rates (*ω*-regimes) without a priori-categorization of the lineage(s) of interest. Additionally, we tested for episodic changes of selection using aBSREL (Smith et al. 2015) in all-to-all branch testing mode.

## Results and Discussion

**Fig. 1.**
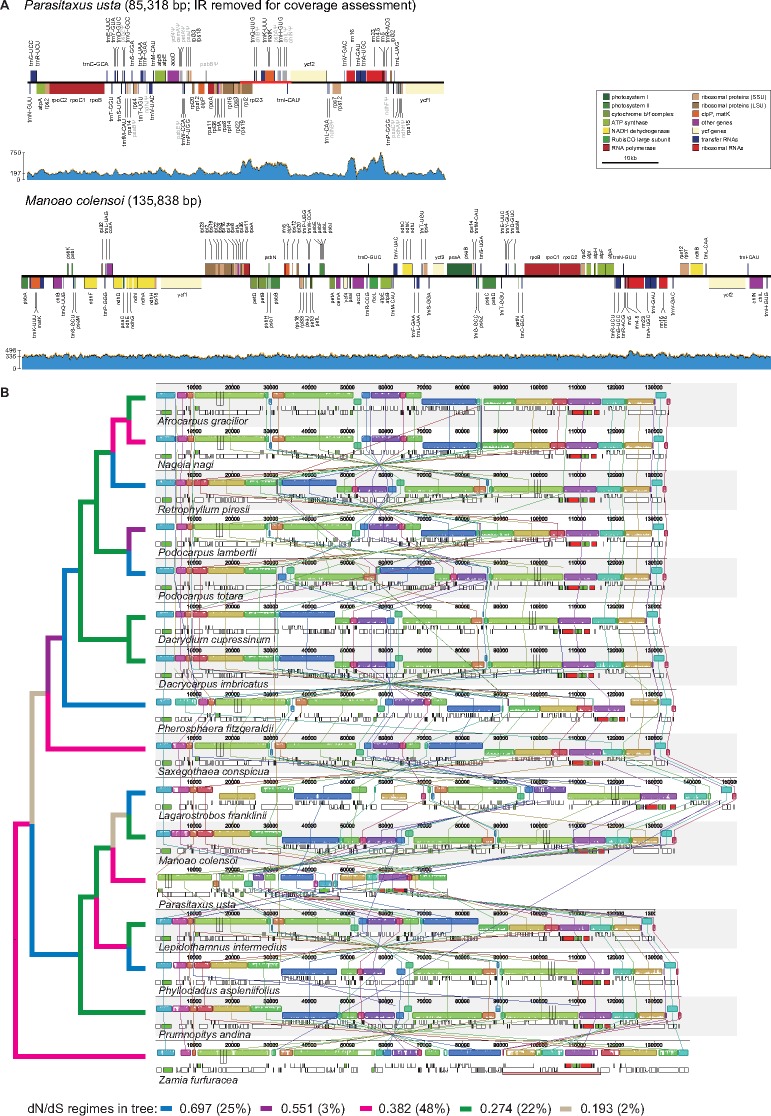
—Plastome structure of *Parasitaxus* in comparison with its autotrophic relatives. (*A*) Linear plastome maps with coverage assessment for the newly sequenced *Parasitaxus* and *Manoao*, drawn to scale. The heterotroph is shown without its IR, which allowed estimating coverage (see supplementary fig. S1, Supplementary Material online, for a full map). One copy of the IRs is highlighted by a red bar. Full-color boxes with labeled gene names highlight coding sequences by gene class, as summarized to the right. Gray text and gene boxes in *Parasitaxus* indicate pseudogenes (*Ψ*). Read depth is scaled by species with the maximum coverage value indicated at the *y* axis. (*B*) The distribution of five selection (*ω*-) regimes within Podocarpaceae is highlighted in the phylogenetic tree, which was inferred by ML from 33 commonly present protein-coding genes. Each branch color represents a distinguished *ω*-class, as indicated at the bottom. Whole-plastome alignments show distinct LCBs as large colored blocks in the upper chromosome drawing per species. Changes in strandedness of LCBs are implied by their illustrations below or above the chromosome bars, and horizontal lines between different species indicate changes of synteny. Sequence conservation within LCBs is indicated by the heights of small bars. The bottom plots per species illustrate the gene distribution in the plastome, where white boxes correspond to protein genes, red to rRNA, and green to tRNA. All plastomes are drawn to scale.

Our two assemblies (Spades, OGA) of the 12 and 74 millions of reads obtained for *Parasitaxus* and *Manoao*, respectively, produced identical results for each of these species’ plastome structures. Coverages above 195× for both species allowed us to build high-quality annotations based on DNA evidence and comparisons with previously sequenced gymnosperms (Chaw et al. 2018; Sudianto et al. 2019). With a length of 85,318 bp (fig. 1*A* and supplementary fig. S1 and supplementary table S1, Supplementary Material online), *Parasitaxus* has the smallest plastome among all sequenced gymnosperms. Unlike other cupressophytes, the plastome of the heterotroph shows a quadripartite structure with an LSC of 38,859 bp, SSC of 27,947 bp, and IRs of 9,256 bp length each. We identified the latter by coverage plots (fig. 1*A* and supplementary fig. S1, Supplementary Material online) and a thorough inspection of read-pair splits, anchoring in the IR on one end but to different single-copy regions on the other.

*Parasitaxus* has lost nearly 60% of the typical gymnosperm plastome coding capacity. Although most Podocarpaceae plastomes, including that of the newly sequenced *Manoao*, encode 82 proteins, 32 tRNAs and 4 rRNAs (Sudianto et al. 2019), *Parasitaxus* retains only 33 intact protein-coding, 31 tRNA and four rRNA genes (supplementary fig. S1 and supplementary table S1, Supplementary Material online). We detected fragments of missing plastid genes (*ndhB*/*D*/*E*/*K*, *petB*, *psaA*/*B*, and *psbB*) on contigs with extremely low (<1) k-mer coverage. This finding suggests that *Parasitaxus* retains (fragmented) copies of these genes in other genomic compartments as nuclear or mitochondrial plastid inserts, similar to parasitic Orobanchaceae (supplementary fig. S4, Supplementary Material online; Cusimano and Wicke 2016).

**Fig. 2.**
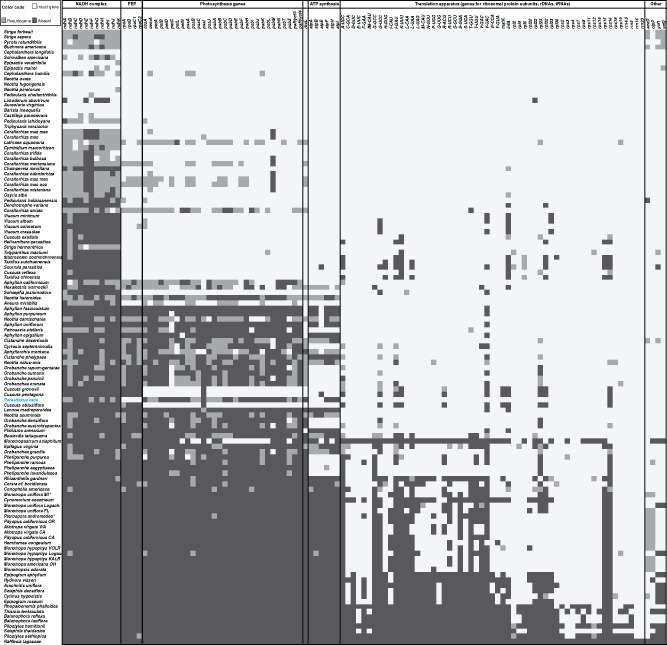
—Plastid coding capacities of *Parasitaxus* and other heterotrophic land plants. The presence and absence of plastid genes across all currently studied plastomes of heterotrophic plants compiled by Wicke and Naumann (2018) and all data published since (full list of included data: supplementary table S4, Supplementary Material online) is depicted for all plastid gene classes. Not included here are the *chl* genes, which have been lost ancestrally in angiosperms, unrelated to heterotrophy (Wicke et al. 2011). *Parasitaxus* is highlighted in blue. Offwhite and light gray indicate the presence of specific genes as intact or pseudogene, whereas the absence of a gene from a plastome is marked in dark gray. Heterotrophic plant species are sorted by decreasing plastome size (from top to bottom).

Notably, gene losses affect almost exclusively photosynthesis genes (49 genes). Only a single tRNA gene (*trnR-CCG*) is undetectable. Thus, the pattern of plastid gene losses in *Parasitaxus* differs from other nonphotosynthetic plants (fig. 2), which even in early stages of genome degradation exhibit functional losses also in plastid ribosomal protein genes or subunits of the plastid-encoded polymerase (Wolfe et al. 1992; Funk et al. 2007; McNeal et al. 2007; Petersen et al. 2015; Logacheva et al. 2016; Naumann et al. 2016; Wicke et al. 2016; Barrett et al. 2018; Su et al. 2019).

Of the plastid-encoded components for photosynthesis, only three (*atpA*/*B*/*E*) of six ATP synthase genes remain in the plastome as intact genes. Their prolonged survival might relate to their proximity to functionally essential plastid genes (Wicke et al. 2013). Sequence comparisons provide circumstantial evidence that 18 other retained photosynthesis genes are nonfunctional (supplementary fig. S2, Supplementary Material online). Of particular interest is the presence of pseudogenes for the light-independent chlorophyll biosynthesis genes *chlB*, *chlL*, and *chlN*. These three genes are typically missing from angiosperm plastomes and have been independently lost in gnetophytes (McCoy et al. 2008; Wu et al. 2009). In contrast, all other gymnosperms (Wu et al. 2007) and nonseed plants, with the exception of a nonphotosynthetic liverwort (Wickett et al. 2008), retain *chl* genes. These results imply that the chlorophyll in *Parasitaxus* (Feild and Brodribb 2005) is a product of the light-dependent chlorophyll biosynthesis pathway or an as yet unknown regulatory system. Trace amounts of chlorophyll and low expression of nuclear-encoded chlorophyll biosynthesis genes were also detected in holoparasitic broomrape (Orobanchaceae) (Wickett et al. 2011) and some orchids (Barrett et al. 2014). Investigating the physiological role of chlorophyll in nonphotosynthetic plants, therefore, may represent a new route of heterotrophic plant research.

All but one plastid gene losses in *Parasitaxus* can be attributed to its transition to heterotrophy. Although genes for the dehydrogenase complex (*ndh* genes) are functionally lost in some gymnosperms (Chaw et al. 2018), all *ndh* genes are intact in photosynthetic cupressophytes. *Parasitaxus* has functionally or physically lost all *ndh* genes (fig. 2 and supplementary table S1, Supplementary Material online). The pseudogenization of all genes for the cytochrome *b_6_/f* complex, the photosystems I and II, and genes directly or indirectly involved in photosynthetic energy conversion explains the observation that *Parasitaxus* plastids lack photoelectron transfer (Feild and Brodribb 2005).

The set of functionally retained protein genes of *Parasitaxus* evolve under purifying selection (fig. 1*B*). We inferred five different *ω*-regimes for Podocarpaceae, all of which <1. *Parasitaxus* belongs to the predominant *ω*-regime (*ω* = 0.382), which describes 48% of the analyzed phylogenetic tree. We found no evidence for episodic changes of selection in the heterotroph or other gymnosperms (all *P* values > 0.55 after alpha error-correction). Together, these data suggest that the transition to heterotrophy might have had a rather mild effect on functionally still relevant plastid genes in *Parasitaxus*. A possible lifestyle effect on the selectional regimes of single genes remains to be investigated.

Our reconstruction of a quadripartite plastome structure for *Parasitaxus* is surprising, given that all studied autotrophic cupressophytes (Wu and Chaw 2014; Qu et al. 2017) have lost their large IRs (fig. 1*B* and supplementary table S1, Supplementary Material online). However, the IRs of *Parasitaxus* differ from the canonical IRs of cycads, ginkgo, and gnetophytes by both their size and gene composition (Guo et al. 2014). Besides being less than half the size of other gymnosperm IRs, the heterotroph’s large repeats contain *trnI-CAU*, *trnH-GUG*, *matK*, *trnK-UUU*, *trnQ-UUG*, *rpl23*, *rpl2*, and *rps19* (fig. 1*A*); all typically located in the LSC. We also detected coverage spikes higher than those of the novel IR in the 16S and 23S rRNA genes, which normally reside in canonical IR regions. Typically, these genes are GC-richer than the rest of the plastome. So, compositional-related genomic fragmentation biases might have caused uneven sequencing. We also cannot exclude the possibility that mitochondrial reads cross-mapped to the plastome despite high-stringency mapping. However, no even coverage spans the plastid rDNA region and no read-pair splits to different single-copy regions exist. Thus, we interpret the coverage spikes as technical artifacts and not as evidence for canonical IRs.

An IR pair is thought to be essential for stabilizing plastome structure (Marechal and Brisson 2010). Inversions often characterize IR-lacking plastomes (fig. 1*B*), as seen in Podocarpaceae (Sudianto et al. 2019). As *Parasitaxus* is nested within the latter (supplementary fig. S3, Supplementary Material online), we conclude that the heterotroph must have gained novel IRs after its divergence from the IR-lacking rest. To us, secondary IR gain is the most parsimonious explanation, more likely than independent losses. Regaining a large IR is exceptional, reported to date only once before in the legume *Medicago minima* (Choi et al. 2019). Choi et al. (2019) hypothesize that IR reemergence could occur through synthesis-dependent strand annealing or the formation and resolution of Holiday junctions during recombination-dependent DNA repair.

Achlorophyllous plants exhibit structural rearrangements beyond gene loss-related syntenic changes (e.g., Delannoy et al. 2011; Bellot and Renner 2015; Feng et al. 2016). We observe an abundance of simple sequence repeats and repeats in the plastome of *Parasitaxus* (supplementary tables S2 and S3, Supplementary Material online), potentially relevant for the development of simple sequence repeat markers to study the evolutionary history of this vulnerable parasitic gymnosperm. *Parasitaxus* also shows unique rearrangements compared with its close relatives (fig. 1*B*). However, in the heterotroph’s plastome, the association between changes in genomic structure and its lifestyle is coincidental (LRT *P* value: 0.862). Rearrangements are prominent in plastomes of Podocarpaceae (fig. 1) and might relate primarily to other lineage-specific factors. Phylostatistical testing cannot support the hypothesis that IR absence and the number of rearrangements are linked in Podocarpaceae (LRT *P* value: 0.531). Similarly, neither an association between the absence of an IR and repeat density (LRT *P* value: 0.935) nor correlations between the latter and the transition to heterotrophy (LRT *P* value: 0.936) or with rearrangements (LRT *P* value: 0.411) exist in Podocarpaceae (supplementary table S3, Supplementary Material online). Typically, these factors are associated with one another in nonparasitic as well as in parasitic plant plastomes (Ruhlman and Jansen 2014; Wicke and Naumann 2018). Thus, our results suggest that plastome evolution in Podocarpaceae and *Parasitaxus* follows unparalleled routes, worthy of further study.

## Supplementary Material

Supplementary data are available at *Genome Biology and Evolution* online.

## Supplementary Material

evz187_Supplementary_Data

## Notes

**Data deposition:** This project has been deposited at GenBank under the accession MN016935 and MN016936.
