# Supplementary material for: Plastome Reduction in the Only Parasitic Gymnosperm Parasitaxus Is Due to Losses of Photosynthesis but Not Housekeeping Genes and Apparently Involves the Secondary Gain of a Large Inverted Repeat
Source: Genome Biol Evol. 2019 Aug 27;11(10):2789–96. doi: 10.1093/gbe/evz187 (PMC6786476; doi:10.1093/gbe/evz187)
Supplement: evz187_Supplementary_Data [file evz187_supplementary_data.zip › Figure_S1-S4.pdf]

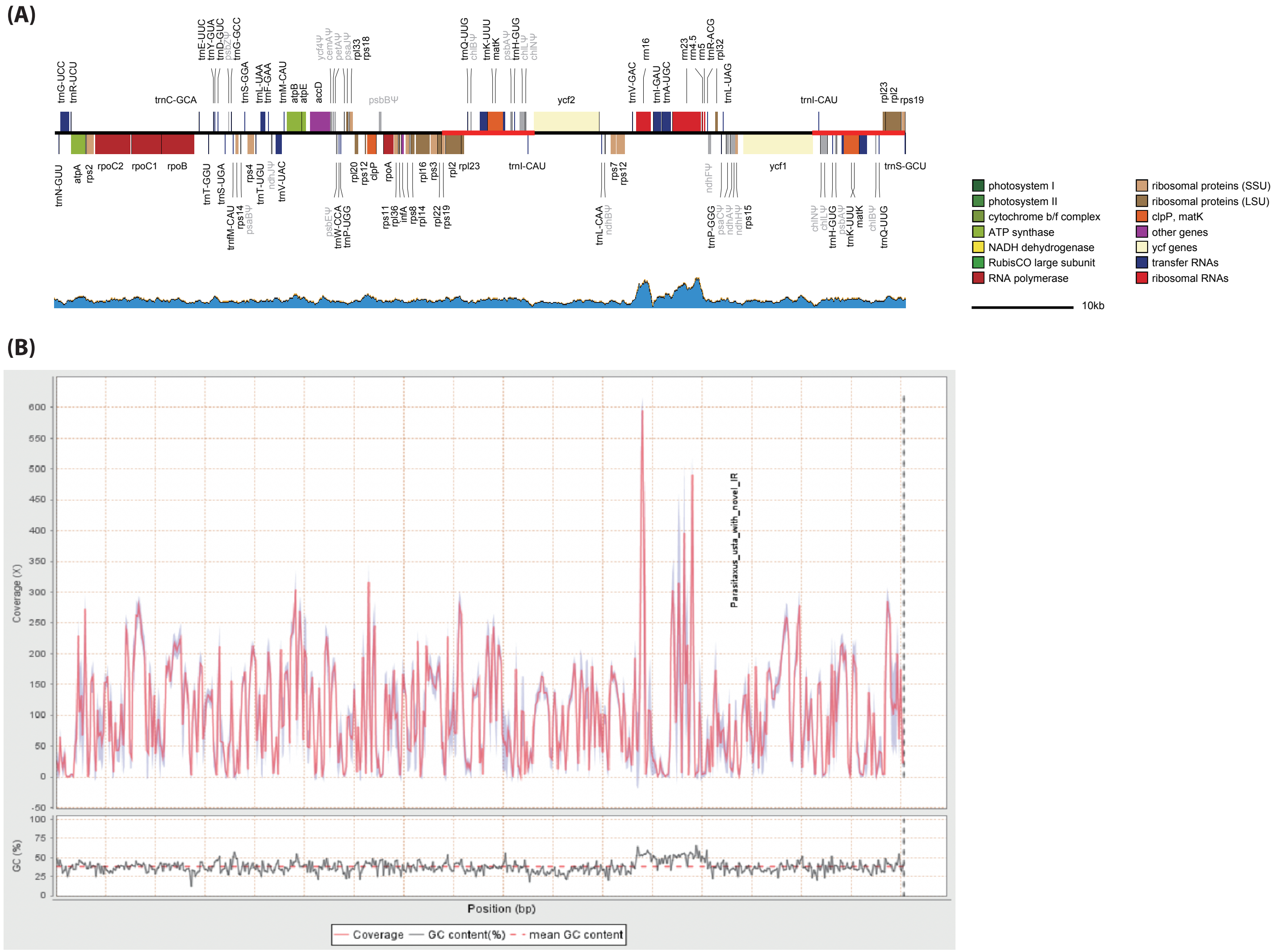

Fig. S1. Full-length plastome map and read coverage distribution for *Parasitaxus*. (A) Linear plastome map for the complete *Parasitaxus* plastome, drawn to scale. The read coverage distribution is indicated below for the entire plastid chromosome. Full-color boxes with labeled gene names highlight coding sequences by gene class, as summarized to the right. The IRs are highlighted with a red bar. Gray text and gene boxes indicate pseudogenes (Ψ). **(B).** Aligned to the plastid chromosome shown in (A), the coverage is shown in relation to GC content, suggesting that the extreme coverage spikes around the *rrn* genes are associated with the above-average GC content of the plastid rRNA genes.

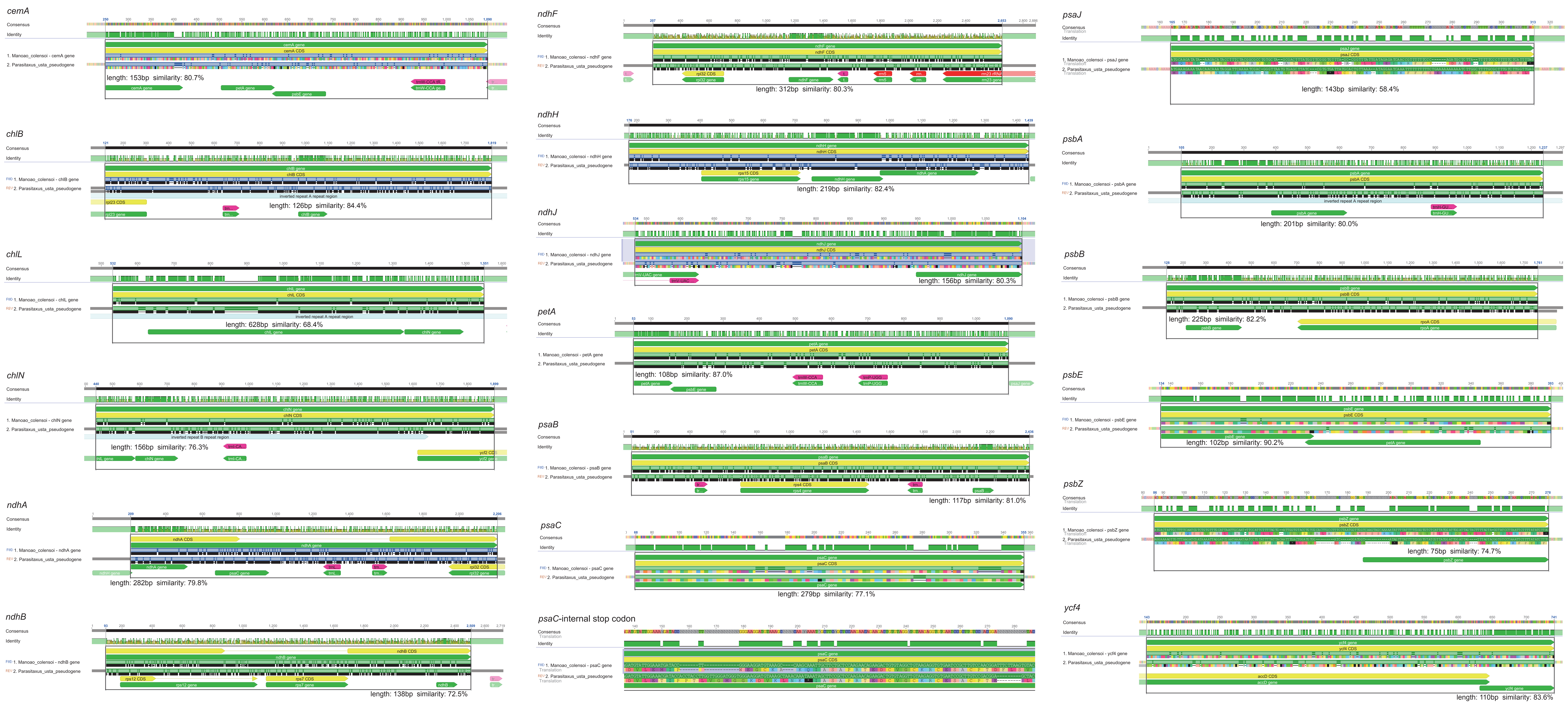

Fig. S2. Pairwise alignment of pseudogene candidates of *Parasitaxus* with intact genes of *Manoao*. Intact genes from the *Manoao* plastome are indicated in both green (gene) and yellow (CDS), whereas the corresponding pseudogene candidate of *Parasitaxus* is indicated only in green (gene). A green bar in the identity row at the top of each gene plot means that nucleotide positions are identical between both species, and yellow indicates differences.

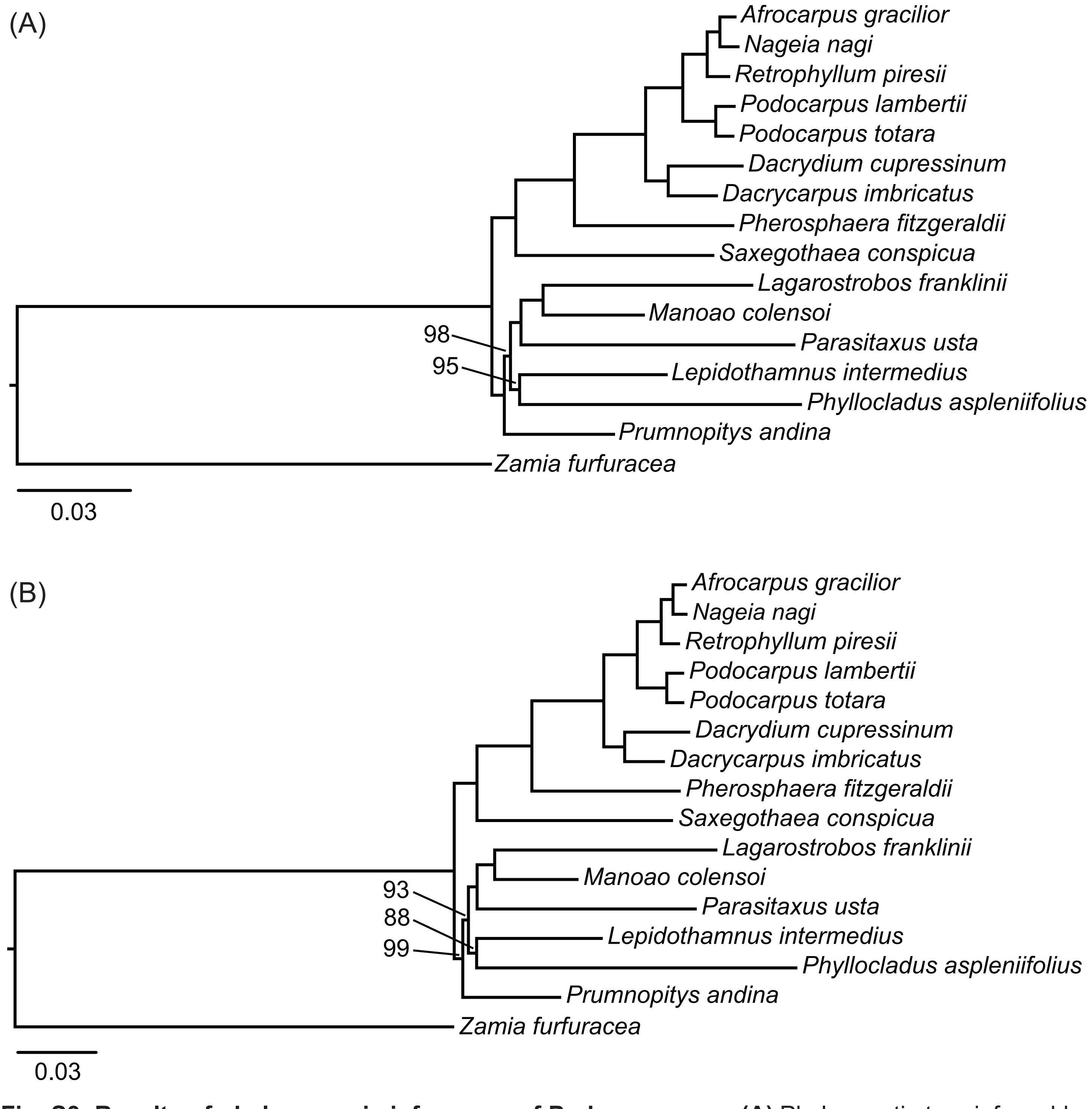

Fig. S3. Results of phylogenomic inferences of Podocarpaceae. (A) Phylogenetic tree inferred by ML using a data set of 118 genes. **(B)** The phylogenetic tree resulting from an ML inference based on 68 commonly present genes. Numbers at branches are bootstrap values, where node support was under 100%.

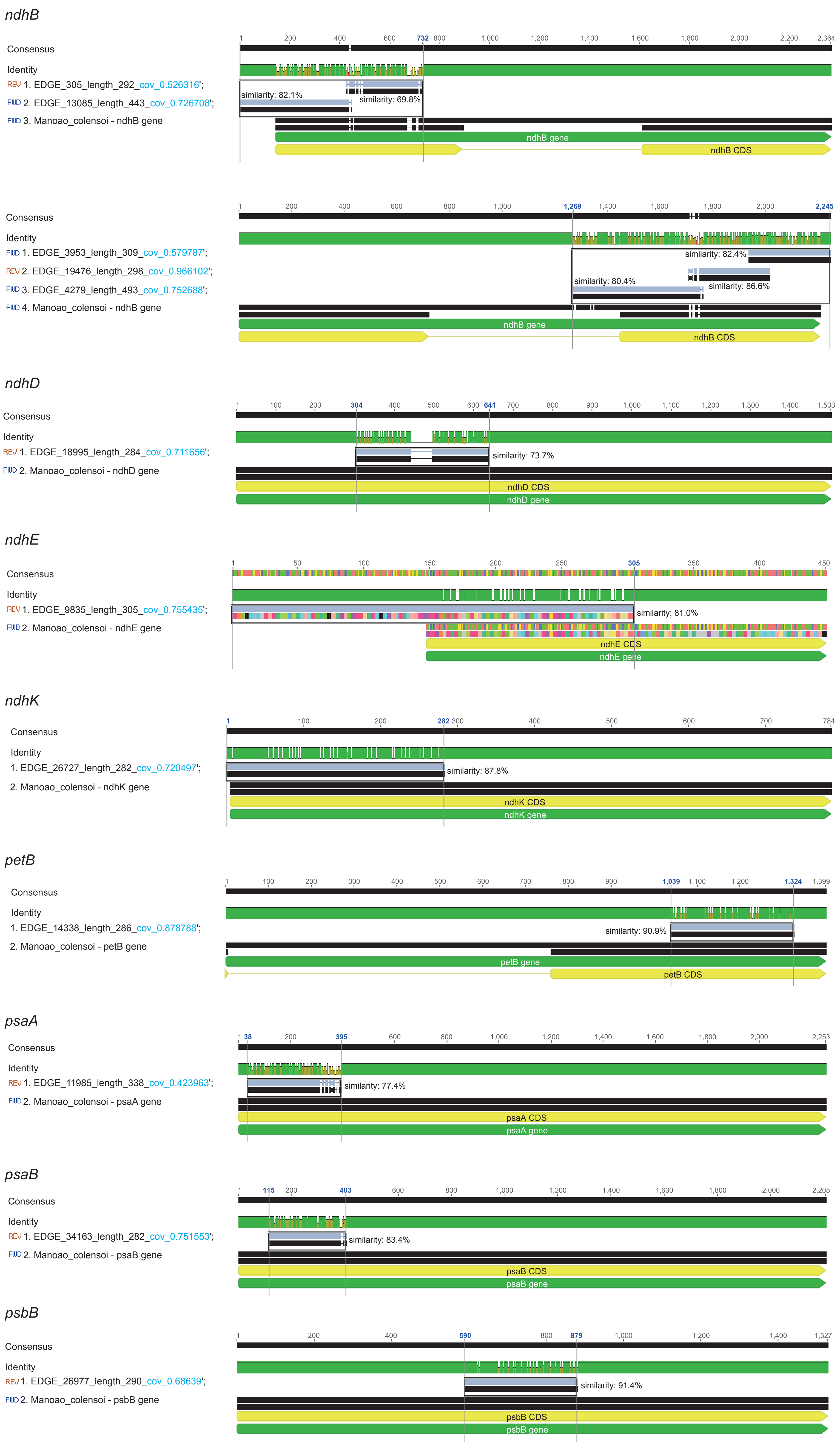
ndhB
Consensus
Identity
REV 1. EDGE_305_length_292_cov_0.526316';
FWD 2. EDGE_13085_length_443_cov_0.726708';
FWD 3. Manoao_colensoi - ndhB gene
similarity: 82.1%
similarity: 69.8%
ndhB gene
ndhB CDS
Consensus
Identity
FWD 1. EDGE_3953_length_309_cov_0.579787';
REV 2. EDGE_19476_length_298_cov_0.966102';
FWD 3. EDGE_4279_length_493_cov_0.752688';
FWD 4. Manoao_colensoi - ndhB gene
similarity: 82.4%
similarity: 80.4%
similarity: 86.6%
ndhB gene
ndhB CDS
ndhD
Consensus
Identity
REV 1. EDGE_18995_length_284_cov_0.711656';
FWD 2. Manoao_colensoi - ndhD gene
similarity: 73.7%
ndhD CDS
ndhD gene
ndhE
Consensus
Identity
REV 1. EDGE_9835_length_305_cov_0.755435';
FWD 2. Manoao_colensoi - ndhE gene
similarity: 81.0%
ndhE CDS
ndhE gene
ndhK
Consensus
Identity
1. EDGE_26727_length_282_cov_0.720497';
2. Manoao_colensoi - ndhK gene
similarity: 87.8%
ndhK CDS
ndhK gene
petB
Consensus
Identity
1. EDGE_14338_length_286_cov_0.878788';
2. Manoao_colensoi - petB gene
similarity: 90.9%
petB gene
petB CDS
psaA
Consensus
Identity
REV 1. EDGE_11985_length_338_cov_0.423963';
FWD 2. Manoao_colensoi - psaA gene
similarity: 77.4%
psaA CDS
psaA gene
psaB
Consensus
Identity
REV 1. EDGE_34163_length_282_cov_0.751553';
FWD 2. Manoao_colensoi - psaB gene
similarity: 83.4%
psaB CDS
psaB gene
psbB
Consensus
Identity
REV 1. EDGE_26977_length_290_cov_0.68639';
FWD 2. Manoao_colensoi - psbB gene
similarity: 91.4%
psbB CDS
psbB gene
