# Supplementary material for: Plastome Reduction in the Only Parasitic Gymnosperm Parasitaxus Is Due to Losses of Photosynthesis but Not Housekeeping Genes and Apparently Involves the Secondary Gain of a Large Inverted Repeat
Source: Genome Biol Evol. 2019 Aug 27;11(10):2789–96. doi: 10.1093/gbe/evz187 (PMC6786476; doi:10.1093/gbe/evz187)
Supplement: evz187_Supplementary_Data [file evz187_supplementary_data.zip › Supporting_Information_Legends.pdf]

Supplementary Figures and Tables

Fig. S1. Full plastome map for *Parasitaxus*. Linear plastome map of the complete *Parasitaxus* plastome, drawn to scale, and coverage distribution over the entire chromosome. The IRs were highlighted with a red bar. Full-color boxes with labeled gene names highlight coding sequences by gene class, as summarized to the right. Gray text and gene boxes indicate pseudogenes (Ψ).

Fig. S2. Pairwise alignment of pseudogene candidates of *Parasitaxus* with intact genes of *Manoao*. Intact genes from the *Manoao* plastome are indicated in both green (gene) and yellow (CDS), whereas the corresponding pseudogene candidate of *Parasitaxus* is indicated only in green (gene). A green bar in the identity row at the top of each gene plot means that nucleotide positions are identical between both species, and yellow indicates differences.

Fig. S3. Results of phylogenomic inferences of Podocarpaceae. (A) Phylogenetic tree inferred by ML using a data set of 118 genes. **(B)** The phylogenetic tree resulting from an ML inference based on 68 commonly present genes. Numbers above branches are bootstrap values, where node support was under 100%.

Fig. S4. Pairwise alignment of nuclear/mitochondrial plastid DNA fragments of *Parasitaxus* with intact genes of *Manoao*. Intact genes from the *Manoao* plastome are indicated in both green (gene) and yellow (CDS), whereas the corresponding nuclear/mitochondrial plastid DNA fragments of *Parasitaxus* is indicated only in shaded blue. The length and kmer coverage of each fragments are shown on the left, and the latter is shown in blue. A green bar in the identity row at the top of each gene plot means that nucleotide positions are identical between both species, and yellow indicates differences.

Table S1 Details of physical features and gene content of plastomes from 15 Podocarpaceae species and *Zamia*.

Table S2 Simple sequence and tandem repeats in the plastome of *Parasitaxus usta* and *Manoao colensoi*

Table S3 Plastome trait data as used for phylogenetic hypothesis testing

Table S4 Summary of data and literature used to compare plastid gene contents between *Parasitaxus* and different lineages of heterotrophic plants shown in Figure 2.
